# Pharmacists at the frontline beating the COVID-19 pandemic

**DOI:** 10.1186/s40545-020-00210-w

**Published:** 2020-04-20

**Authors:** Nadia Bukhari, Huma Rasheed, Bismah Nayyer, Zaheer-Ud-Din Babar

**Affiliations:** 1 School of Pharmacy, University College London, WC1N 1AX, London, UK; 2 Institute of Pharmaceutical Sciences, University of Veterinary and Animal Sciences, Lahore, Pakistan; 3 Department of Population Health Sciences, Faculty of life Sciences & Medicine, King’s College London, London, SE1 1UL UK; 4 Department of Pharmacy, University of Huddersfield, Queensgate, Huddersfield, Huddersfield, HD1 3DH UK

## Abstract

As the lockdowns are being observed all over the globe and the national level pharmacy professionals are performing frontline roles, this editorial highlights the role of pharmacists in the COVID − 19 pandemic. Pharmacists globally are providing services amidst pandemic, including TRIAGE services, seeing patients and reducing the patients’ burden on health care facilities such as hospitals and GP practices. Pharmacists are also working to providing home deliveries, as well as dealing with the increasing number of patients coming through to pharmacies with the other ailments. Pharmacy associations have issued their guidelines and in this editorial, several global examples of pharmacists’ role in the COVID 19 are being discussed. Pakistan is used as a country case study in this editorial. The editorial also elaborates how pharmacists in the UK and Pakistan have teamed up together to compile 10-steps protection guidelines for the pharmacy teams in Pakistan in English and Urdu language. This 10-point guidance educates community pharmacies for safety and standard operation as the number of patients in the country continues to rise. These guidelines are endorsed by the government and private bodies. These can be adopted and adapted by any country; keeping in view their laws and regulations.

## Background

### Pharmacists and Covid-19 pandemic

Through public preventative measures advocated by WHO, the public are working together in their respective countries to ‘flatten the curve’. With a near enough global lockdown there seems to be an even greater dependence on pharmacists as the first point of contact to fulfil the public’s healthcare needs. Pharmacies around the world are one of the few places that are kept open for public service even during the strict lockdowns [1].

Community pharmacists and their teams are a vital healthcare provider during the outbreak; they remain on the frontline of public health by serving as direct points of access for their patients. Hospital pharmacists have an important role during the outbreak in infection control as well as patient care and support [2].

Countries severely hit by pandemic are exceedingly facing overburdened Health facilities and shortages as well as burnouts of health care professionals. TRIAGE service has emerged as a supportive modality in this time of crisis which includes pharmacists along with other primary health care workers [3]. A provision of COVID-19 trained health care professionals exists for supporting these services in the time of shortage of medical and nursing staff members and to increase the outreach of the service in Australia as well. The International Pharmaceutical Federation (FIP) has issued a pack of 10 summaries for guidance on COVID-19 [4]. American Pharmacist Association (APhA) has also issued guidelines and resource documents for the strengthening and preparedness of the community pharmacies as front line health care workers in the global health crisis [5].

Reliability of information and control of scare and misinformation are important concerns during the worldwide spread of the disease. Community pharmacists also continue to play their role towards public uninterrupted for regular supplies of medicines, as well as supporting governments [6] for disseminating information on precautions related to COVID-19 spread including hand washing technique to availability of face masks and instructions for their proper use and disposal. USP has issued guidelines for the compounding and pharmacists and manufacturers for preparation of hand sanitizers to cope with the stock outs [7].

The community pharmacy has a unique credible role with ease in accessibility. In France, the campaign against domestic violence also involved the use of the code “Mask 19” to report domestic violence by the victims [8]. Pharmacists are an integral component of healthcare performing extraordinary roles in the earlier pandemics and health crisis, with some like Ebola and Zika posing global health security risks as well [2]. Likewise, by contributing in the prevention, preparedness and response to COVID-19 pandemic community pharmacists are delivering their role towards public health in dealing with this crisis [9]. In many countries, pharmacies have worked in close collaboration with the International humanitarian organizations like Red Cross and local community workers to increase its outreach to public and ensure home delivery of medicines [10].

In New Zealand, the pharmacist’s contribution is appreciated by the government by extra remuneration for their support. Hotline numbers have been issued for encouraging on phone consultation and prescription orders for home supplies to reduce public visits which are to be avoided in case of suspected and confirmed COVID cases [11]. It is encouraged for pharmacies in Australia to support remote dispensing of prescriptions using prescriptions received through mails/faxed/emailed or making use of electronic transfer of prescriptions (ETP) technology couples with home delivery services in particular for the old and vulnerable populations [12].

### China, Colombia and elsewhere

Lower Middle-Income Countries (LMIC) are in a greater need for pharmacists’ support where patients are unable to afford the doctor fees for a consultation. In absence of a standard treatment the importance of provision of pharmaceutical care by pharmacists managing the COVID-19 cases increased many-folds [13]. Similarly a different set of attributes then the routine conditions were observed in the pharmaceutical care provided by the Chinese Community pharmacists in dealing with the pandemic situation including maintaining controlled work environment, provision of information and necessary medical supplies as well as ensuring the regular medication and counselling are managed at best to avoid undue patient visits to healthcare facilities. The Community pharmacists in China were made use all possible resources to perform their role as care providers and custodian of patient safety regarding medicine use. They used Mobile applications, coordination with neighbourhood committees and medicine companies to ensure delivery of medicines to patients in their homes [10]. The response to COVID-19 pandemic in countries have differed drastically. In Columbia, a lesser incidence of hospitalization and no intensive care need was attributed to better preparedness, higher exposure to multiple strains of viral respiratory infections as well as early adoption of containment strategies [14].

### Pakistan and COVID-19

As of 6 April, there are 3059 confirmed cases & 45 deaths with COVID-19 in Pakistan [15]. Since the first case reported on 26 Feb 2020, Pakistan has put in place its mitigation strategies which are framed around suspension of flight operations both international and domestic, social distancing, point of entry screening, contact tracing, clinical management of COVID-19 patients, Home Quarantine and Isolation & burial procedures for COVID-19 patients [16] To ramp up the efforts of containing the spread of COVID-19, the federal government imposed a partial lockdown on 21 March 2020 in which schools and pupil places are closed. Public transport is suspended. Public Hospitals are turned into isolation wards and the working class has been advised to work from home. Bakeries, Utility Stores, vegetables shops and dairy shops are opened but that too between specific hours. People can come outside of their homes for essential trips and emergencies only [17]. After re-evaluating the situation based on increasing cases within Pakistan, federal government has extended the partial lockdown on 1 Apr 2020 for two more weeks [18].

### Economical effects re-aligning the access to pharmacies during COVID-19 crisis in Pakistan

The response to this COVID-19 pandemic in the shape of social distancing requiring country lockdown and close of businesses, is a death sentence to the daily wagers and low working class in Pakistan. According to experts the number of people who can lose their jobs in various sectors are between 12.3 million and 18.5 million [19]. Although, Pakistan as a fragile and indebted economy has come up to the rescue of its vulnerable people with a multi-trillion-rupee relief package, allocating 200 billion rupees for the labour class. Under the package, some 10 million people, categorized under low-income groups, will get a lump sum amount of 12,000 PKR, initially for a period of four months in the form of Ehsaas Emergency Cash Program [20]. However, this amount is lower than the daily wages they were getting before lockdown [19]. These economically pressured masses don’t have much to spend on their health especially when the public hospitals have closed their OPDs for the general public. With no were to go and no money to spend, they access their nearby pharmacies which can get them medical advice without paying for consultation and they only have to pay for the medicine if it’s needed. This could be a great window to showcase the role of community pharmacists in LMICs particularly during a time of a health emergency.

### Telemedicine, pharmacists and Pakistan

With the closure of OPDs in the private and public hospitals, there has been a great unrest among the patients with other diseases. With nowhere to go and to exercise and save themselves from the virus exposure at the hospitals, opens the door for Telemedicine provisioning in this time of crisis. There is a growing demand for telehealth and telemedicine has already been in use Pakistan for a while [21]. With the doctors, nurses and paramedics physically present in the emergency, isolation wards and quarantine centers, pharmacists can hold onto this end and can ease the patient distress by providing triaging and basic consultations via Telemedicine, taking the burden off the doctors and health system.

### Development of the COVID-19 pharmacy guidelines in Pakistan

In Pakistan, majority of the pharmacists are engaged with the pharmaceutical industry, however there is a growing number which is now working in the community pharmacies or at retail pharmacies. While the organizations, authorities, hospitals were arranging personal protection equipment (PPEs) for doctors, nurses and paramedics, little attention was being paid towards the protection of pharmacy teams even though they are the first line of contact for the public in normal situation and even more in this COVID-19 pandemic with closing of public OPDs. Assessing this scenario worldwide, Pharmacists in the UK and in Pakistan team up together to compile 10-steps protection guidelines for the pharmacy teams in Pakistan in English and Urdu language. These guidelines are shown in Table 1.

**Table 1:** COVID-19 10 Step guidance for pharmacy teams

The features of the guidelines are:	
1. Pharmacy Signage: Have a banner/standee at the pharmacy entrance advising patients not to enter the pharmacy if they are displaying any signs or symptoms of COVID-19. Signpost patients to contact the COVID-19 Helpline 1166 or to contact COVID-19 designated hospitals	
2. Wash your hands: Regularly wash your hands with soap and water for at least 20 s or use an alcohol-based rub. Use WHO 7 steps of hand-washing technique. Provide Hand gels at the pharmacy counter for the public.	
3. Self-Isolate: If you have a new cough and/ or a fever DO NOT come to work and self isolate for at least 14 days and when symptoms get better	
4. Social Distance: Maintain a 1-m distance between yourself and patients when taking in and giving out prescriptions. Consider restricting the number of patients who can enter your pharmacy at one time	
5. Face mask: Wear a mask when in contact with patients. Change masks frequently. Disposable masks should only be used once.	
6. Prescription Handling: Wear disposable gloves in the pharmacy. Ensure you change your gloves every time you handle a new prescription	
7. Mobile Phones Rx Handling: Encourage patients to sanitize mobile phones with alcohol wipes available at the pharmacy counter, before you handle the mobile	
8. Cash/PC Handling: Cash should be strictly handled with gloves and should be changed after every hour. Handling of medicines and cash should not be done by the same member of staff simultaneously. Use gloves to operate keyboards	
9. Sanitation of Premises: All surfaces including appliances, shelving, medicines packaging, computers, telephones should be sanitized on a regular basis using a rota-system	
10. COVID-19 Testing: will only take place if:	
● Recently travelled internationally	
● Recently travelled Intercity and are showing COVID-19 Symptoms	
● Been in-contact with someone who has recently travelled Internationally	
● Anyone displaying COVID-19 symptoms: fever, persistent cough & Shortness of breath	

The COVID-19 guidance for pharmacy teams in Pakistan was endorsed by the Ministry of National Health Services, Regulation and Coordination (M/o NHSR&C) and supported by The International Pharmaceutical Federation (FIP), The Commonwealth Pharmacists Association (CPA), Drug Regulatory Body Pakistan (DRAP), Pakistan Pharmacists Association (PPA), one of Pakistan’s leading telemedicine providers – doctHERS, The National Alliance of Women in Pharmacy, Pakistan (NAWP) and Pharmacy Council Pakistan.

These guidelines can be adopted and adapted by any country keeping in view their own laws and regulations.
